# Application of Innovative Technologies for Improved Food Quality and Safety

**DOI:** 10.1155/2016/9160375

**Published:** 2016-01-04

**Authors:** Yiannis Kourkoutas, Nikos Chorianopoulos, Aspasia Nisiotou, Vasilis P. Valdramidis, Kimon A. G. Karatzas

**Affiliations:** ^1^Applied Microbiology and Molecular Biotechnology Research Group, Department of Molecular Biology and Genetics, Democritus University of Thrace, 68100 Alexandroupolis, Greece; ^2^Institute of Technology of Agricultural Products, Greek Agricultural Organization Demeter, 14123 Athens, Greece; ^3^Department of Food Studies and Environmental Health, Faculty of Health Sciences, University of Malta, Msida 2080, Malta; ^4^Department of Food and Nutritional Sciences, University of Reading, Reading RG6 6AD, UK

Nowadays, consumers in developed countries demand food products of high-quality, which are easy-to-handle, safe, and with natural flavor and color, as well as with an extended shelf life [1]. One of the most significant challenges in the food industry is to apply efficient technologies in the production chain to fulfill these demands. Major studies have introduced innovative technologies, such as nonthermal or alternative, quicker, and sensory-milder thermal methodologies [2]. Methods that are used today, such as packaging, application of antimicrobials (natural or chemicals), pasteurization, high pressure processing, or combinations of these technologies (hurdle technologies), have been widely explored and introduced applicable control tools in reducing bacterial contamination levels [1, 2].

In this respect, application of new discoveries in health-promoting food manufacture has been an intriguing and exciting topic of major public concern, while at the same time it confers multiple advantages in the industrial sector. In response to demands from increasingly health conscious consumers, the global new trend focuses on foods not only with superior sensory appeal but also of improved nutritional attributes. Current innovations in food production have resulted in the development of novel products with specific functional ingredients, reduction or removal of undesirable components, modification of food compositions, and overall improved quality and safety.

One of the main objectives of this special issue was to provide a number of documents to summarize new inventions, developments, and ideas in the most recent innovation opportunities and technological achievements in the production of foods with potent beneficial effects. In this context, the structural characteristics and antioxidant activities of soy protein isolate (SPI) dextran conjugates obtained by TiO_2_ photocatalysis treatment were explored. Results revealed that the UV-vis absorption and the fluorescence intensity increased with higher photocatalytic power. SPI-dextran conjugate was successfully formed by TiO_2_ photocatalysis treatment. Likewise, the effect of chitosan coating with cinnamon oil on the physiological attributes and preservation quality of China jujube fruits during storage was investigated. This investigation revealed that the complex coating of chitosan and cinnamon oil could directly control disease decay and maintained the good sensory acceptability of jujube fruits. Moreover, during storage time, the treatment of chitosan-oil coating might maintain the quality attributes and induce the defense reaction system of jujube fruits.

A universal approach based on “omics” technologies for the quality control of food was fully discussed. Up-to-date technologies based on digital information systems such as web platforms and smartphone applications, can facilitate the adoption of molecular labelling. In this respect, recent smartphone “apps” are becoming a powerful tool to promote the consumption of high-quality foodstuffs and in particular the consumption of those food items able to prevent diseases [3–6]. Such informative tools (including online portals and dissemination web sites) can be useful for different stakeholders to translate a molecular label based on “omics” in a more understandable language for the whole category of consumers.

An overview of current and new approaches in genetically modified organisms detection was also assumed. Furthermore, the benefits and drawbacks of such methodologies were discussed, too. In addition, a protocol was validated and determined to be suitable for practical use in monitoring and identifying genetically modified rice. The results indicated that the established methodology is convenient, rapid, and of low-cost for routine detection of genetically modified rice.

Finally, nanotechnology has proven its competence in almost all possible fields to food and agricultural systems [7–9]. Hence, a summary of the different methods of food processing, packaging, and preservation techniques is provided and the role of nanotechnology in the food processing, packaging, and preservation industry is highlighted and assessed.

*Yiannis Kourkoutas* *Nikos Chorianopoulos* *Aspasia Nisiotou* *Vasilis P. Valdramidis* *Kimon A. G. Karatzas*
